# Glutamine Homeostasis and Its Role in the Adaptive Strategies of the Blind Mole Rat, Spalax

**DOI:** 10.3390/metabo11110755

**Published:** 2021-10-31

**Authors:** Dmitry Miskevich, Anastasia Chaban, Maria Dronina, Ifat Abramovich, Eyal Gottlieb, Imad Shams

**Affiliations:** 1 Department of Evolutionary and Environmental Biology, Faculty of Natural Sciences, University of Haifa, Haifa 3498838, Israel; anastasia@chaban.su; 2 Institute of Evolution, University of Haifa, Haifa 3498838, Israel; dronina.maria@gmail.com; 3 Technion Faculty of Medicine, Haifa 3525433, Israel; ifat.a.g@gmail.com (I.A.); e.gottlieb@technion.ac.il (E.G.)

**Keywords:** glutamine, metabolome, hypoxia, proline cycle, GSH, adaptation, bioenergetics

## Abstract

Oxidative metabolism is fine-tuned machinery that combines two tightly coupled fluxes of glucose and glutamine-derived carbons. Hypoxia interrupts the coordination between the metabolism of these two nutrients and leads to a decrease of the system efficacy and may eventually cause cell death. The subterranean blind mole rat, *Spalax*, is an underexplored, underground, hypoxia-tolerant mammalian group which spends its life under sharply fluctuating oxygen levels. Primary *Spalax* cells are an exceptional model to study the metabolic strategies that have evolved in mammals inhabiting low-oxygen niches. In this study we explored the metabolic frame of glutamine (Gln) homeostasis in *Spalax* skin cells under normoxic and hypoxic conditions and their impacts on the metabolism of rat cells. Targeted metabolomics employing liquid chromatography and mass spectrometry (LC-MS) was used to track the fate of heavy glutamine carbons (^13^C_5_ Gln) after 24 h under normoxia or hypoxia (1% O_2_). Our results indicated that large amounts of glutamine-originated carbons were detected as proline (Pro) and hydroxyproline (HPro) in normoxic *Spalax* cells with a further increase under hypoxia, suggesting a strategy for reduced Gln carbons storage in proteins. The intensity of the flux and the presence of HPro suggests collagen as a candidate protein that is most abundant in animals, and as the primary source of HPro. An increased conversion of αKG to 2 HG that was indicated in hypoxic *Spalax* cells prevents the degradation of hypoxia-inducible factor 1α (HIF-1α) and, consequently, maintains cytosolic and mitochondrial carbons fluxes that were uncoupled via inhibition of the pyruvate dehydrogenase complex. A strong antioxidant defense in *Spalax* cells can be attributed, at least in part, to the massive usage of glutamine-derived glutamate for glutathione (GSH) production. The present study uncovers additional strategies that have evolved in this unique mammal to support its hypoxia tolerance, and probably contribute to its cancer resistance, longevity, and healthy aging.

## 1. Introduction

Glucose (Glc) and glutamine (Gln) are two central metabolic nutrients that maintain cellular metabolism. Gln has a more pleiotropic role in cellular metabolism than Glc. It incorporates directly into proteins [1] and participates in the proteostasis regulation [2]; provides carbons and nitrogen for the biosynthesis of amino acids, nucleotides, and hexosamines [1,3,4]; replenishes the tricarboxylic acid (TCA) cycle with carbons [5]; and plays an important role in the gluconeogenesis (GNG) [6].

Hypoxia upregulates the level of hypoxia-inducible factor 1α (HIF-1α) which switches cellular bioenergetics to anaerobic mode [7]. The anaerobic mode includes reduced pyruvate metabolism, uncoupling of the glycolysis and the TCA cycle, and the subsequent shortage of glucose-derived acetyl-CoA (Ac-CoA) [8,9]. In turn, the deficit of ac-CoA could result in a decreasing citrate (Citr) level that is essential for multiple biosynthetic reactions and signaling [10]. Replenishing of the TCA cycle with Gln-derived carbons, termed Gln anaplerosis (Gln-An), is the only known way to maintain appropriate levels of Citr and anabolic processes in case of a Glc-derived Ac-CoA deficit [8]. Gln-An works independently of Glc entry into the cycle and allows the TCA cycle to function regardless of oxygen availability. Gln-An can furnish the cells’ necessities by several ways, e.g., conversion to malate (Mal) and further to pyruvate (Pyr) via a malic enzyme (ME) reaction thereby maintaining NADPH^+^ level. Pyr can be converted to lactate (Lact) for maintaining the cytosolic NAD^+^ pool and is released outside, otherwise, returns to the TCA cycle via pyruvate dehydrogenase (PDH) for producing Ac-CoA. Oxaloacetate (OAA) is the downstream Mal processing product that serves as a precursor for aspartate (Asp) and asparagine (Asn), or after condensation with Ac-CoA maintains the Citr pool. Hypoxia switches the oxidative Gln metabolism to the reductive mode via ubiquitylation of SIAH2, a subunit of α-ketoglutarate (αKG) dehydrogenase (αKGDH) which is required for the conversion of αKG to succinate (Succ) [11]. Besides the TCA cycle transformations of Gln, its function and metabolism in relation to proline (Pro) biosynthesis is of special interest [12], particularly the use of Gln carbons for Pro and collagen synthesis [13,14]. The metabolism of de novo produced Pro and hydroxyproline (HPro) (as a product of collagen degradation) is suggested as an alternative way for canonical bioenergetics and signaling under hypoxia and starvation conditions for tumor cells. Phang et al. firstly presented redox cycle that couples the pentose phosphates path (PPP) and cycling of Pro and pyrolline-5–carboxylate (P5C) which generates ATP and NADP^+^ in rat kidney mitochondrion, termed the proline shuttle (PS) [15]. Lately, Hagedorn and Phang published direct evidence of PS existence in a cell-free experimental system and described the mechanism of its function [16]. Further investigations of Pro and HPro metabolism shed light on its antioxidant [17], signaling [18,19,20,21], and bioenergetics [22,23] roles in the cellular homeostasis under stress condition. 

The present study uncovers the essentials of Gln homeostasis in primary cells that were isolated from the *Spalax ehrenbergi* complex (*spalacidae*) (hereafter, *Spalax*). *Spalax* is an underground mammal which harbors unique features such as constitutively higher levels of HIF-1α [24], hypoxia tolerance (3% under laboratory conditions, and down to ~7% oxygen recorded in natural habitat [25]), extreme longevity (up to 20 years) [26]; oxidative stress [27]; and cancer resistance [28]. Typically, most of the metabolome explorations were carried on immortal cultured cell lines or tissues that were harvested from laboratory animals. Nevertheless, a few works have characterized the metabolome of wild animals [29,30,31], however, to our best knowledge, there are no studies specifically addressing Gln metabolism. In the present study we aimed to address Gln homeostasis in primary skin cells that were harvested from *Spalax* that were captured in the wild, in comparison with primary skin cells that were taken from a laboratory rat (*Rattus norvegicus*) under regular oxygen content (~20% O_2_, normoxia) and under hypoxia (1% O_2_). A stable isotope labelling approach was chosen to achieve this purpose [32]. Typically, ^13^C_5_ Gln is a tracer of choice for the evaluation of the total contribution of Gln in the TCA cycle and lipogenesis, de novo Pro biosynthesis, and reductive carboxylation (RC) [33,34,35,36]. Cells that were cultured with medium that was enriched with stable-isotope-labeled Gln consume and metabolize it allowing heavy Gln-derived carbons to be integrated into metabolic reactions and appear as heavy metabolites. These metabolites will be designated in the study as the metabolite’s name followed by ‘M+n’ (M is the molecular mass; n is the number of heavy carbons ^13^C incorporated in the metabolite).

## 2. Results

**Figure 1:**
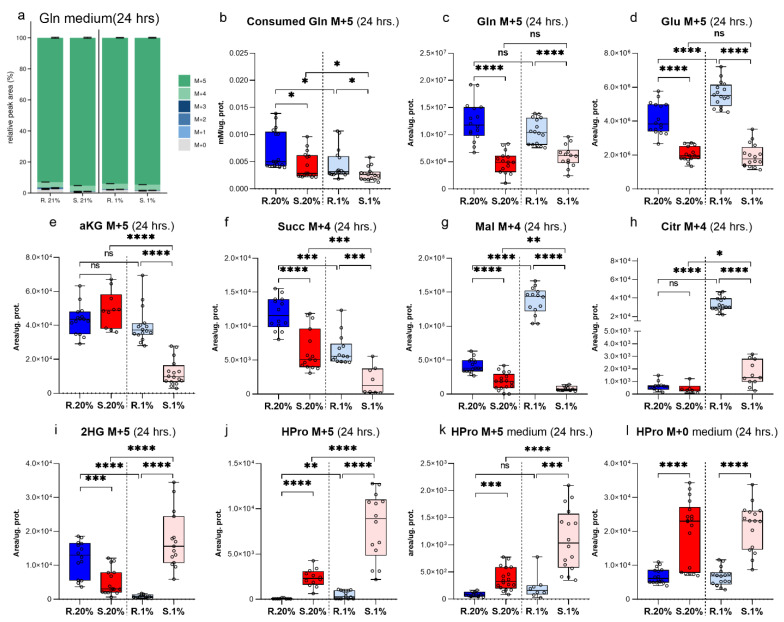
Selected characteristics of Gln homeostasis in the *Spalax* and rat cells after one day under normoxia and hypoxia: (**a**) the MID for medium Gln, (**b**) the Gln M+5 that was consumed by the cells (**c**), (**d**)-intracellular levels of Gln M+5 and Glu M+5; the levels of the TCA cycle Gln-derived intermediates: (**e**–**h**) the levels of intracellular αKG M+5, succinate M+4, malate M+4, citrate M+4, Orn M+5, HPro M+5; and the fate of diverted from the TCA cycle Gln M+5: (**i**,**j**) intracellular levels of 2HG M+5, HPro M+5; (**k**,**l**), secreted outside HPro M+5, HPro M+0. S.20%, R. 20%, S.1%, R1% represents *Spalax* (S) and rat (R) cells that were exposed to an atmosphere containing 20% or 1% O_2_, respectively; ns, (nonsignificant) *p* > 0.05; * *p* < 0.05; ** *p* < 0.01; *** *p* < 0.001; **** *p* < 0.0001, the error bars represent standard deviation of six or more biological repeats. Every point on the chart represents one technical repeat.

The distributions of heavy Gln carbons through metabolic landscape after one day of the experiment demonstrates a complete ^13^C enrichment picture. The overall mass isotopologues distribution (MID) for major metabolites of Gln are presented in Supplementary Figure S1. Gln M+5 is a dominant mass isotopologue (MI) in the culture medium (Figure 1a) (~95% of the total pool of Gln is found in the medium), therefore the rest of its MI’s can be neglected. Further, the Gln homeostasis will be described as transformations of consumed heavy Gln M+5 carbons through three sections: glutamate (Glu) and αKG metabolic domains, and reductive Gln metabolism.

*Spalax* cells consumed less Gln than the rat, but metabolized it faster into Glu and further to αKG, glutathione, ornithine, and proteins.

**Figure 2:**
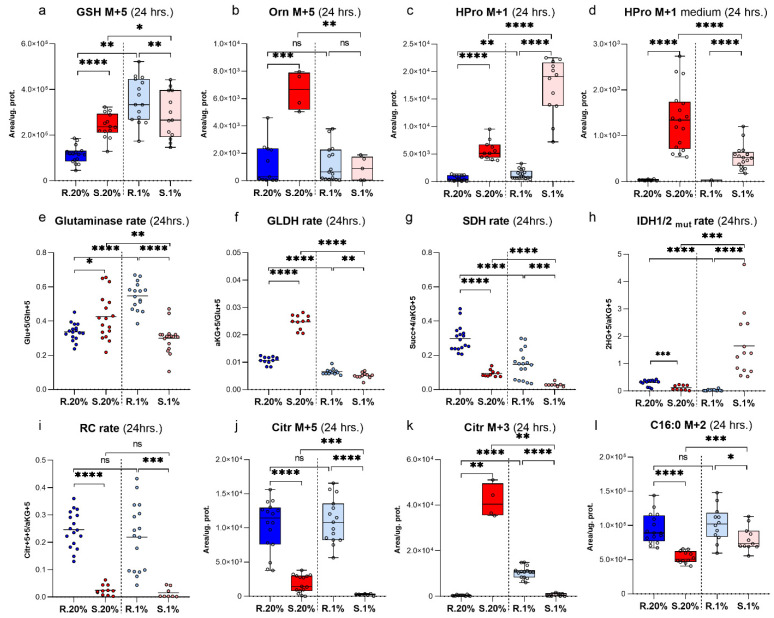
Tracking the fate of Gln+5 after non-TCA cycle transformations: intracellular levels of (**a**) GSH+5; (**b**) Orn+5; The reductive carboxylation of Gln+5; intracellular levels of (**c**) HPro+1; (**j**) citrate+5; (**l**) C16:0+2; (**d**) extracellular levels of HPro+1; the Malic Enzyme-metabolized Gln+5: intracellular levels of (**k**) citrate+3. The calculated rates for (**e**–**i**) glutaminase, glutamate dehydrogenase, succinate dehydrogenase, and isocitrate dehydrogenase in *Spalax* and rat cells that were cultivated for one day under normoxia and hypoxia. S.20%, R. 20%, S.1%, R1% represent the *Spalax* (S) and rat (R) cells that were exposed to an atmosphere containing 20% or 1% O_2_, respectively; ns, (nonsignificant) *p* > 0.05; * *p* < 0.05; ** *p* < 0.01; *** *p* < 0.001; **** *p* < 0.0001, the error bars represent standard deviation of 6 or more biological repeats. Each point on the chart represents one technical repeat.

The consumption of Gln M+5 from the medium by the normoxic rat cells was significantly higher than the *Spalax* (Figure 1b). The consumed Gln M+5 is deaminated by glutaminase (GLS) into Glu M+5 (Figure 1c,d) [37]. The Gln deamination rate was higher in the resting *Spalax* cells compared to the rat (Figure 2e). Interestingly, the GLS activity was significantly higher in *Spalax* cells, while elevated levels of intracellular Glu M+5 were observed in the rat. The Glu domain, besides Glu incorporation into protein, includes the usage of Glu for *de-novo* Pro, ornithine (Orn), reduced glutathione (GSH) biosynthesis, and its conversion to αKG [38]. Thus, in normoxic *Spalax* cells, Glu M+5 flux markedly bifurcates between de novo biosynthesis of Pro M+5, Orn M+5, GSH M+5, and its oxidation to αKG M+5. The newly produced Glu M+5 is distributed between αKG M+5, Orn M+5, GSH M+5, and is detected as intra/extracellular HPro M+5. (Figure 1d,e,j,k and Figure 2a,b, Figure S1k,l,o,p).

The character of Glu M+5 distribution changed under low oxygen conditions in the *Spalax* cells. The significantly decreased levels of αKG M+5 (Figure 1e) and the diminished glutamate dehydrogenase (GLDH) rate (Figure 2f) suggest a rerouting of the Gln-derived carbons flux from αKG to other destinations inside the Glu-domain, such as GSH and protein production (Figure 1j and Figure 2a,k). The increased heavy HPro M+5 and the elevation of non-labeled HPro M+0 in the medium hypoxic *Spalax* cells (Figure 1l and Figure S1p) suggest a turnover of one or more Pro-rich proteins that were also harboring HPro.

### Characteristics of αKG Metabolic Domain 

The αKG domain is a metabolic hub for Gln-derived carbons. αKG was subjected to oxidative metabolism entering the TCA cycle (measured as levels of Succ M+4) (Figure 1f), or reductive carboxylation (displayed as levels of Citr M+5), as well as directly converted to 2HG (observed as 2HG M+5) (Figure 1i).

**Figure 3:**
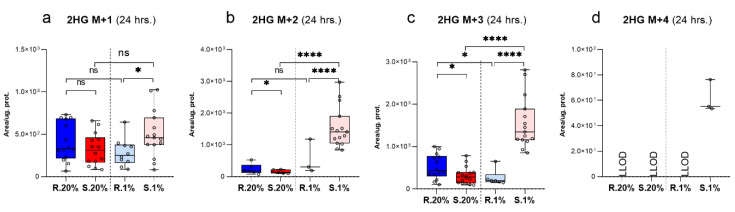
The intracellular levels of different mass isotopologues of 2HG. (**a**) 2HG M+1; (**b**) 2HG M+2; (**c**), 2HG M+3; and (**d**) 2HG M+4 in the *Spalax* and rat cells under normoxia and hypoxia after they were cultivated for one day under normoxia and hypoxia. S.20%, R. 20%, S.1%, R1% represent the *Spalax* (S) and rat (R) cells that were exposed to an atmosphere containing 20% or 1% O_2_, respectively; ns, (nonsignificant) *p* > 0.05; * *p* < 0.05; **** *p* < 0.0001, the error bars represent the standard deviation of six or more biological repeats. Every point on the chart represents one technical repeat. LLOD-lower limit of detection.

GLDH mediates the conversion Glu to αKG [39]. The rate of GLDH under normoxic conditions in the *Spalax* cells was significantly higher than the values that were calculated for the rat cells (Figure 2f). The metabolic transformations of αKG in the normoxic rat cells seemed to be much more active compared to *Spalax*. Newly produced αKG M+5 in rat cells more dynamically entered the TCA cycle as Succ M+4 (Figure 1f–h) as compared to *Spalax*, metabolized to 2HG M+5, or alternatively subjected to reductive metabolism (detected as Citr M+5) (Figure 1f–i and Figure 2j). The RC-originated Citr M+5, after its shuttling to the cytosol, cleaves with ATP citrate lyase (ACLY) into M+3 and M+2 fragments. The M+3 fraction actively metabolizes in the *Spalax* cells compared to the rat cells; it is processed via phosphoenolpyruvate carboxykinase (PEPCK) losing one carbon, enters the glycolysis as phosphoenolpyruvate (PEP M+2), and sinks as Ala M+2, or is processed with PDH and appears as intro/extracellular HPro M+1 (Figure S2(Aa) and Figure 2c,d). In contrast, in the normoxic rat cells, an upstream GNG flux of M+3-originated Gln carbons was indicated (Figure S2(Ac,d)). The only active path in the *Spalax* cells compared to the rat cells was the shuttling of Gln-originated carbons between the mitochondria and the cytosol. We observed an ME-driven process of conversion of mitochondrial Mal M+4 (Figure 1g) to cytoplasmic Pyr M+3 (Figure S2(Ab)) and further returned them with PC assistance as mitochondrial Citr M+3 (Figure 2k). The ME reaction requires NADP, therefore the shuttling aims to maintain an appropriate NADPH pool in the *Spalax* cells. Both other possible directions of metabolic utilization of αKG (RC, normal anaplerosis, and its direct conversion to 2HG) were depressed in the normoxic *Spalax* cells. The frame of αKG metabolic domain in the *Spalax* cells under hypoxia was similar to normoxia: RC and TCA cycle transformations were depressed compared to those in the rat cells. The levels of Citr M+5 measuring the reductive Gln metabolism in hypoxic *Spalax* cells were significantly less than the values that were observed under normoxia (Figure 2j). Interestingly, the transformation of αKG M+5 to Citr M+4 increased in both the hypoxic *Spalax* and rat cells compared to normoxia (Figure 1e,h). In contrast, the upregulated TCA cycle transformation of Citr M+4 to Mal M+4 was detected in the rat cells only (Figure 1g). The newly produced Citr M+4 in the *Spalax* cells was probably shuttled to the cytosol with the citrate shuttle instead of processing in the TCA cycle reactions. On the other hand, the direct conversion rate of αKG M+5 to 2HG M+5 in the *Spalax* cells was strongly elevated compared to both species’ normoxic values and the hypoxic rat cells (Figure 1i and Figure S1i). Hypoxia rerouted the cytosolic fluxes of RC-derived Gln carbons in the *Spalax* cells. The lipogenesis (detected as M+2 C16:0) (Figure 2l) and GNG (appeared as intracellular M+2 MI’s of GAP, DHAP) (Figure S2(Ac,d)) were increased compared to normoxia. The transport of RC-originated M+3 MI via PC to the mitochondria was significantly diminished (measured as Citr M+3 levels) (Figure 2k). The PDH metabolism of cytosolic Gln-derived Pyr M+2 (measured as Citr M+1), and further, the flux to intra/extracellular HPro+1 was upregulated in the hypoxic *Spalax* cells (Figure 2c,d). Notably, regardless of the pathway of their metabolic transformation, Gln-originated carbons appeared as MI’s of 2HG (M+1; +2; +3; +4) exclusively in the hypoxic *Spalax* cells (Figure 3 and Figure S1i).

**Figure 4:**
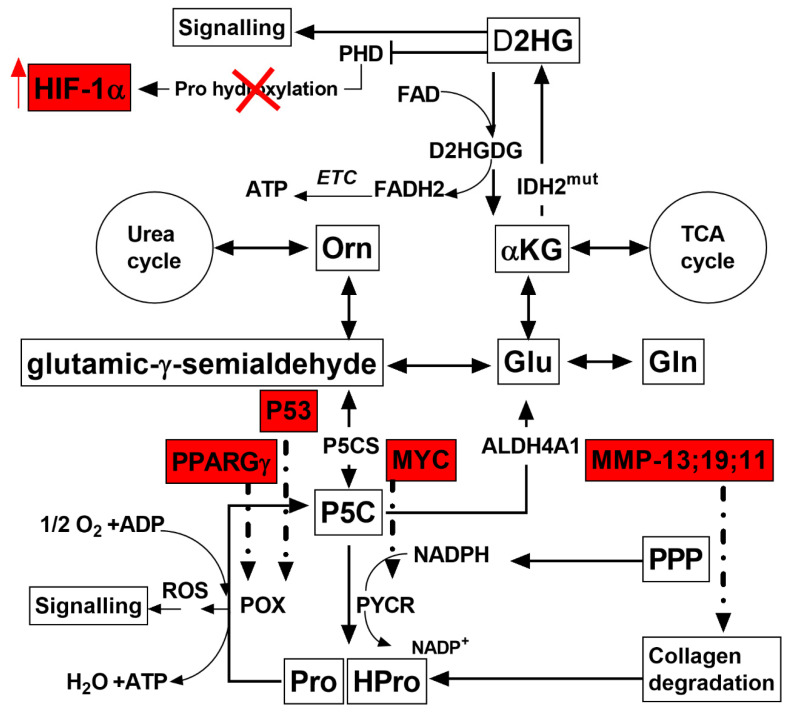
Primary scheme of redox shuttling of Pro/HPro and D2DGDH in the *Spalax* cells. Red-colored abbreviations and lines represent significantly upregulated mRNAs in the liver tissues of *Spalax* cells compared to the rat cells. IDH2^mut^, mutant IDH is known to facilitate 2HG production; nonetheless, the *Spalax* gene (NCBI Reference Sequence XP_008844432.1) does not harbor these mutations/substitutions.

An upregulated expression P53, PPARɣ, and POX genes was observed in the *Spalax* under both normoxia and hypoxia, (Figures S2h,f and S5 and Figure 4). PYCR is another enzyme that plays a role in the proline shuttle which demonstrated an increased gene expression in the liver (Figure S2e). Notably, the POX expression was higher in the *Spalax* fibroblasts, while ALDH18 expression was remarkably higher in the *Spalax* liver (Figure S5). Overexpression of MMP-13, MMP-19, and MMP-11, involved in the breakdown of extracellular matrix (e.g., collagenolysis) was also marked in the present study (Figure S2j–l and Figure 4). 

## 3. Discussion

Glutamine (Gln) is the most prevalent free amino acid in mammalian tissues, blood, and cells. It participates in both anabolic and catabolic processes. Along with glucose Gln is an abundant source of reduced carbon for oxidative metabolism [40]. Most of the Gln is consumed in the glutaminolysis pathway of Gln oxidation to pyruvate, which is involved in energy production and maintenance of the NADPH pool via ME [41]. The final product of glutaminolysis, pyruvate, is a fuel molecule that enters the TCA cycle through PC or as Ac-CoA that is transported into the mitochondria via PDH. This canonical way of utilizing the Gln carbon is a component of central carbon metabolism that is optimal for ATP production. Nevertheless, it is tightly balanced with glycolysis and is controlled via genetic, post-translation modifications, and allosteric mechanisms [42]. The machinery of central carbon metabolism is a fine-tuned system that integrates Glc and Gln carbon fluxes, and ultimately requires an appropriate level of oxygen to allow for oxidative phosphorylation (OXPHOS). It was hypothesized in the present study that in response to low oxygen environment exposure throughout its lifetime [25], *Spalax* evolved an alternative metabolism of Gln that is less dependent on oxygen availability. The metabolic landscape of *Spalax* cells is compared to that of rat cells as an example of canonical central carbon metabolism. 

The framework of glutaminolysis in the *Spalax* cells looks different when compared to rat cells. Both of the cells increased their Gln consumption during the experiment; however, the *Spalax* cells consumed less Gln. Along with Gln consumption, the *Spalax* cells had an elevated deamination rate, thereby introducing Gln carbons to their metabolism faster. Interestingly, a significant fraction of the consumed heavy Gln-derived carbon in normoxic *Spalax* cells was distributed between the metabolites of the Glu domain. The incorporation of Glu M+5 into the structure of GSH (GSH M+5), as well as its direct conversion to Pro M+5 (measured as its hydroxylated form-HPro M+5) and Orn M+5, dominated specifically in the *Spalax* cells (Figure 1j,k, Figure 2a,b and Figure S3A). These pronounced fluxes suggest that the metabolic domains are extremely important for the maintenance of *Spalax* metabolic pattern. The elimination of reactive oxygen species (ROS) is vital for cells that are under hypoxic conditions. Hypoxia and especially sharp periodic changes from hypoxia to oxygenation, which are likely to occur in the *Spalax* underground environment, are well described as inducers of ROS production [43,44]. GSH is well known as an abundant intracellular antioxidant [45]. GSH, as a cofactor of GSH-peroxidase, participates in a non-specific reduction of hydroperoxides resulting in the formation of its oxidized form (GSSG). Thus, GSSG levels indirectly indicate the intensity of the intracellular free radical processes and reflects the balance between the reduced and oxidized forms of glutathione. The glutathione pool in the *Spalax* cells was significantly more reduced than in the rat cells (Figure S3(Ag)) and has the appropriate potential to cope with oxidative stress. It seems, therefore, that *Spalax* cells maintain a massive intracellular GSH pool to prevent oxidative damage. 

The specific rewiring of Glu metabolism to Pro biosynthesis was reported after the reprogramming of human dermal fibroblasts into hepatocyte-like cells [46]. The formation of 1-pyrolline-5-carboxylic acid (P5C) is the hub of the path from Glu to Pro. At the P5C point, Glu-originated carbon has two directions for metabolism: processing via ornithine aminotransferase (OAT) to Orn, or via proline oxidase (POX) to Pro [47]. The elevated de novo production of Orn M+5 that was observed in the *Spalax* cells (Figure 2b) suggests a link to polyamine metabolism. We did not measure the level of polyamines in the current study. However, the levels of the expression of ornithine decarboxylase (ODC), a key enzyme of polyamine metabolism [48], and antizyme inhibitor (AZIN1), a positive regulator of polyamine levels [49], were evaluated in the previously published *Spalax* and rat liver transcriptome data [50] (Figure S2(Bc,f)). A strong upregulation of conversion of Glu M+5 to HPro M+5 was observed in the *Spalax* cells after 24 h (Figure 1j,k). De novo synthesized Pro M+5 was apparently incorporated into Pro-rich protein/s, and was detected as HPro M+5, a product of protein degradation. Collagen is suggested as a primary candidate for such protein [51]. Notably, collagens, which are the most abundant proteins in animals, are well described as the primary source of proline and, particularly, hydroxyproline [52] (and therein cited literature). Other proteins, such as elastin and HIF-1α, may also be involved. Although elastin contains a high percentage of proline (but not HPro), it is known as a durable protein with a unique stable structure that doesn’t undergo significant turnover in healthy tissues where it can last for tens of years [53]. HIF-1α contains hydroxylated proline residues and has a faster turnover; however, as a transcription factor that exists in relatively small amounts, it is unlikely to be a major source of Pro/HProline for proline shuttle. 

Gln-derived carbons enter the TCA cycle after conversion of Glu to αKG by glutamate dehydrogenase (GLDH). The GLDH conversion rate in normoxic *Spalax* cells during the study was similar to that of glutaminase (GLS); the more Glu that was subjected to deamination, the more it was converted to αKG (Figure 2e,f). However, the TCA cycle downstream processing of αKG to Succ decreased in the normoxic *Spalax* cells due to the diminished SDH rate (Figure 2g). Interestingly, in the *Spalax* cells, the flux of Gln-derived carbon after entering the TCA cycle bifurcates at Mal M+4 point (Figure 1g) where one path follows cycle’s reactions to αKG M+4 (Figure 1e), converts to Glu M+4 and appears in the GSH structure as GSH M+4 (Figure S3(Ad)), while the other path metabolizes via ME where glutamine carbons appear as cytosolic Pyr M+3 (Figure S2(Ab)). Apparently, this Pyr M+3 returns via PC to the mitochondria and appears as Citr M+3 (Figure 2k) and following metabolic transformations it is secreted outside the cell as Glu M+3 (Figure S3(Bc)). Likely, after one turn of the TCA cycle circulation, Gln carbons undergo cataplerotic efflux as Glu M+3 from the *Spalax* cells under normoxia.

Glutaminolysis is an oxygen-dependent pathway which is not favored in low oxygen conditions. In hypoxic conditions some cells, such as glioblastoma [54], MRC5 cells [55], HBEC30 and HCC4017 [56] switch to reductive Gln metabolism (RC), while some cells use RC routinely in non-hypoxic conditions [57,58,59,60]. In RC, Gln-derived carbon flux forwarded from αKG directly reduces to citrate instead of being oxidized to succinate. RC machinery under hypoxia is characterized by activity of NADP^+^-dependent IDH1 and is regulated by HIF-1α-dependent mechanism that diverts Gln carbon to FA biosynthesis [55]. The metabolic mastering by HIF-1α includes uncoupling of the glycolysis and the TCA cycle [61], thus RC is a compromise to maintain Gln catabolism under hypoxia. The appearance of Citr M+5 as a result of direct reduction of αKG M+5 by IDH1 (Figure 1e and Figure 2j,h) and specific lipogenesis, i.e., heavy C:16 M+2 (Figure 2l), a consequence of Citr M+5 cleavage, are the signs of RC. RC was observed in cells of both animal species under normoxia. Surprisingly, RC was pronounced in the rat cells as compared to *Spalax*. 

Uniquely for the *Spalax* cells, the OAA M+3 that appears after the breakdown of cytosolic RC-derived Citr M+5 enters the central glycolysis as PEP M+2, returns to the TCA cycle, diverts along with the axis αKG M+1→Glu M+1→Pro M+1 and is finally detected as intra/extracellular HPro M+1 (Figure 2c,d). This supports the hypothesis that collagen biosynthesis is a prioritized pathway of Gln-derived carbon metabolism in *Spalax* skin cells. 

These data suggest that normoxic *Spalax* cells metabolize, most of Gln-derived carbons in the Glu-domain. Gln carcass is mainly used for GSH production or is diverted to Orn/Pro biosynthesis. *De-novo* produced Pro forwards to massive synthesis of HPro-rich proteins. Gln carbon skeleton enters the TCA cycle as αKG incorporates into GSH after one cycle or being subjected to ME shuttling and effluxes as Glu. Both cells employ reductive Gln metabolism under normoxia. However, normoxic *Spalax* cells use reductive Gln metabolism to a lesser extent than rat cells and divert returned after RC carbons for protein synthesis.

The lowering of oxygen concentrations is accurately sensed by HIF-1. HIF-1 induces specific metabolic reprogramming that facilitates adaptation and survival of cells under hypoxia. HIF-1 is described as heterodimer consisting of two subunits HIF-1α and HIF-1β [62]. The regulatory activity of HIF-1 proteins is determined by stability of its α-subunit. Under normoxic conditions, HIF-1α is constitutively degraded; whereas under low oxygen it stabilizes and translocates into the nucleus where it orchestrates the gene expression [63]. The proteasome degradation of HIF-1α is initiated by αKG–dependent prolyl hydroxylase (PHD) via hydroxylation of its Pro residues [64]. The hypoxic metabolic landscape is characterized by decreased levels of total αKG (sum of areas of all αKG MI’s) in both cells compared to normoxic values, while in *Spalax* cells, it remains significantly less than in the rat (Figure S2(Bb)). Thus, the observed αKG shortage can negatively influence on PHD activity in *Spalax* cells. A significant portion of newly consumed Gln M+5 in hypoxic *Spalax* cells after its deamination is directly converted to 2HG by D2HGDH (Figure 1i), similar to the process reported by Struys, E. A et al. for cultured lymphoblasts [65]. This is confirmed by the upregulated rate of IDH 1/2 and increased levels of 2HG M+5 in hypoxic *Spalax* cells as compared to the rat (Figure 1i and Figure 2h). Remarkably, nearly all cytosolic and mitochondrial pathways of Gln metabolism re-divert to 2HG production under hypoxic conditions in *Spalax* cells (Figure 3). The 2HG is a structural analog of αKG which may inhibit αKG-dependent PHD and prevents HIF-1α degradation; therefore, it stimulates its accumulation [66]. Both decreased level of the essential participant of PHD reaction αKG and upregulated production of PHD inhibitor 2HG eventually lead to the stabilization of HIF-1α and its steep increase in *Spalax* cells compared to rat cells. These suggestions agree with the previously observed higher levels of HIF-1α and its target (Epo) in *Spalax* kidney in vivo compared to rat under hypoxia [24]. This proposes the importance of HIF-1α constitutive expression for *Spalax* metabolism. Thus, rewiring of Gln flux in hypoxic *Spalax* cells can be involved in the development of hypoxic HIF-1α-mastered phenotype as an additional factor of HIF-1α reciprocal stabilization. 

αKG and 2HG form a redox couple that may have some bioenergetics implications and could play a role in the development of specific epigenetic landscape in *Spalax* cells under hypoxia. 2HG is oxidized to αKG by mitochondrial D-2-hydroxyglutarate dehydrogenase (D-2HGDH) [67]. 2HG oxidation uses specific machinery involving FAD and an electron acceptor that was identified later by Struys et al. as a membrane-associated flavoprotein ubiquinone oxidoreductase (ETFQO) [68] that transfers electrons to ETC [69,70]. Transcriptome data showing that liver expression of both 2HGDH and ETFQO is much higher in *Spalax* as compared to the rat (Figure S2(Bm,n)) support the idea proposed here about the energetic outcome of the redox pair αKG and 2HG. 

Another implication of the raised 2HG production that merits further investigation is the specific epigenetic landscape in *Spalax* cells. The inhibition of αKG-dependent oxidases causes alterations in hydroxylation, demethylation, halogenation, desaturation, epoxidation, ring forming reactions, etc. [71]. This may lead to specific changes in the epigenetic modifications; nevertheless, this subject requires further investigations.

Cells of both animal species reduce the rate of αKG entry into the TCA cycle under hypoxia, but the conversion of αKG M+4 to Succ M+4 as well as glutaminolysis are significantly more reduced in *Spalax* cells compared to the rat cells (Figure 1e,f and Figure 2e,g). Probably, the deficit of pyruvate-derived Acetyl-CoA, which is a consequence of HIF-1α-mediated inhibition of PDH [61], is the reason for the decreased carbon flux via the TCA cycle reactions that was observed in our study. Surprisingly, the RC is not activated in *Spalax* cells under hypoxia but actively engages in rat cells (Figure 1d).

Interestingly, hypoxic *Spalax* cells use a similar metabolic framework which was observed under normoxia, namely, significantly upregulated cataplerosis of consumed Gln carbon skeleton for protein biosynthesis. The heavy Gln carbon fluxes, regardless of the followed metabolic path (mitochondrial or cytosolic), return to the mitochondria where Gln carbon skeleton is used for synthesis of Pro and is likely incorporated into collagen in which Pro/HPro (Figure 1j–l, Figure 2c,d and Figure S1k,l) accounts for about 30% of amino acid residues [72,73]. The specific role of collagen as a stress substrate is reported by Phang et al. [74]. Authors figuratively defined collagen as a “sink for reducing potential that can be easily and reversibly removed from the metabolic pool” [74]. The massive carbon flux (seemingly into collagen production) observed in *Spalax* cells in both normoxic and hypoxic conditions suggests that collagen may play an essential role in *Spalax* metabolism. Collagen is considered as a ‘depot’ for Pro and HPro and some other amino acids similar to glycogen for glucose and adipose tissue for fatty acids. The particular importance of collagen is its role as a reservoir for two amino acids Pro and HPro, both of which are involved in the regulation of intracellular redox balance. The discovery of the specific redox role of POX in the degradation of Pro [75] and HPro [76] suggests that these metabolites have a special role as metabolic regulators [77] and as bioenergetic substrates alternative to glucose [16,78,79] in *Spalax* cell metabolism. High HPro levels in *Spalax* cells in normoxia is clearly shown by the present results, and their further upregulation under hypoxia suggests the involvement of these metabolites in cell bioenergetics as it was described by Phang et al. [80] who proposed ‘Proline shuttle’ as an original mechanism of redox shuttling of Pro and HPro which provides several bioenergetic advantages to cells. 

The final step of Pro biosynthesis is cytosolic reduction of ∆^1^pirolline-5-carboxylate (P5C) by P5C reductase (PYCR) using NADPH as electron donor. This reaction is reversed by mitochondrial POX, which uses FAD as a prosthetic group and directly reduces Cytochrome-C in the ETC. Thus, the cycling of Pro in the proline shuttle allows transport of cytosolic reducing equivalents from NADPH into mitochondria with certain energetic outcome (~1.5 ATP per one FADH molecule) [16,81]. FAD, being part of POX, reduces oxygen to superoxide when exposed to dissolved intracellular oxygen instead of passing electrons to the ETC. Thus, the proline shuttle function is also responsible for ROS generation involved in the regulation of oxidative defense, apoptosis [82], senescence [18] and hypoxic response [83]. The switch between ROS production and passing electrons to the ETC in the POX takes place via moving the flexible a-helix domain of the enzyme to permit access to dissolved oxygen (ROS mode) or to block it (ETC mode) [74]. 

Interestingly, the proline shuttle (Figure 4) is not regulated by HIF-1α, as reported by Phang et al. POX, induced by the p53 gene [74], belongs to the family of 14 genes that are more than seven-fold expressed in response to the induction of P53 and designated as “PIGs” by Polyak et al. [84]. Another POX inducer is peroxisome proliferator-activated receptor Gamma (PPARɣ) [74]. Upregulated expression of both genes-P53; PPARɣ as well as POX was observed in *Spalax* compared to rat in both normoxia and hypoxia [50], (Figure 4, Figures S2h,f and S5). Other enzymes, participants of the Proline shuttle, PYCR and P5C synthase (P5Cs), are regulated by expression of oncogene MYC that controls conversion of Glu to Pro and its induction positively correlates to PYCR1 and P5Cs activity [83]. The marked overexpression of MYC in liver transcriptome [50] of *Spalax* compared to rat is in good agreement with the increased expression of PYCR1 in the liver (Figure S2e). Also, the expression of proline-metabolizing enzymes in skin fibroblasts and liver tissues of *Spalax* and rat under normoxia and hypoxia was evaluated using q-PCR approach. Interestingly, in tested skin fibroblasts the POX expression is significantly higher in *Spalax*, while the liver specifically upregulated ALDH18 expression, the enzyme converting Glu to P5C, and POX/PYCR maintaining redox coupling between Pro/HPro and P5C (Figure S5).

Pro circulation in the proline shuttle can be easily terminated, and carbon skeleton may get further metabolized as Glu or Pro. De novo produced Pro can be secreted from the cell and reabsorbed back when necessary [85], or used intracellularly. Collagen is an essential source of Pro. Collagen from extracellular matrix (ECM) is easily degraded by a family of matrix metallopeptidases (MMP) [86]; however, according to experimental data reported by Imberman et al., a greater part of collagen produced de novo by fibroblasts in culture degrades intracellularly before its secretion from the cell and is detected as HPro [51]. Increased levels of HPro detected in our study in both intra- and extracellular space in *Spalax* compared to rat is clear evidence of facilitated collagen metabolism. The importance of collagen degradation in the *Spalax* metabolism is supported by overexpression of specific MMP-13, MMP-19 and MMP-11 which are involved in the breakdown of extracellular matrix (collagenolysis) (Figure S2j–l). Increased Pro and HPro production in tumor cell lines in response to hypoxia is regulated by HIF-1α [87]. Similarly, hypoxia-induced upregulation of HPro levels was observed in *Spalax* cells. Free HPro is not a proteinogenic amino acid [88], and its enzymatic degradation is performed by the same machinery that is used for Pro degradation [89]. Therefore, both free Pro and HPro can be metabolized by the Proline shuttle and considered as an alternative bioenergetic substrate for survival under hypoxic stress. 

Two factors have probably influenced the development of the metabolic pattern of *Spalax* cells, hypoxia and diet. *Spalax* spends most of its life underground in self-constructed burrows system and accumulates food mainly from the underground sources. *Spalax* is a strict vegetarian. We have found food storages in *Spalax* nests that were full mostly of root pieces, bulbs, local flora’s leaves, and stalks during multiple field expeditions. *Spalax* individuals used for the study were captured from the Lower Galilee. The traditional ancient crop of this land is a chickpea (*Cicer arietinum*) [90],which roots (visually defined) have found in the food storage of *Spalax*. With a high degree of probability, the roots of *Fabaceae* family plants are a major meal for *Spalax*. The essential feature of Fabacea plant’s roots is their ability to symbiotic nitrogen fixation by rhizobium [91] and high production of nitrogen-rich amino acids such as Gln [92] and Asn [93]. Seemingly, the Gln and Asn-rich ration and lesser/sporadic availability of Glc was a trigger to evolving the specific bioenergetics mode. The thin diet during evolution processes have forced to shape the metabolic systems with a deficient of some specific vital compounds. For example, we observed lower levels of niacine, nicotinamide, pathothenate, folate, riboflavin (Figure S4) in *Spalax* cells compared to the rat. The deficit of these metabolites may seriously influence on the metabolic machinery in general and pyruvate dehydrogenase complex in particular, and therefore influences/interrupts/changes its machinery. The combination of thin diet and permanent low oxygen conditions may influence pyruvate dehydrogenase complex (PDC) and limit its functions as a metabolic hub of central carbon fluxes in *Spalax* cells. Therefore, due to specific environmental conditions evolutionary processes may have compromised the role of PDC in *Spalax* evidenced in our study. Another aspect that probably had influenced the evolution of *Spalax* cells’ metabolic model is periods of starvation. The tunneling activity of *Spalax* is limited to the rainy period when the soil is wet and can be easily excavated. This period (about 4–5 months) is the only time for building new galleries, mating, annual food harvesting and storage. Drought, common in Middle East, possesses starvation challenge for this mammalian group. Glycogen and fat are emergency tools for fixing glucose starvation for most mammals. Seemingly, when Glc is less accessible than glutamine, the accumulation of Glc carbons as glycogen or converting them to fat is less advantageous than the storage of Gln-derived carbons as extracellular collagen suggested in the present study. 

## 4. Materials and Methods

### 4.1. General Experimental Design

The work was performed on cultured primary fibroblasts and on tissues harvested from *Spalax* and rats. For metabolic studies, cells were treated by hypoxia (1% O_2_) and compared to normoxic cells. For gene expression analyses, total RNA was extracted from hypoxic and normoxic animal tissues (6%, or 21% O_2_, respectively) RNA was subjected to cDNA synthesis and quantitative real-time PCR. 

### 4.2. Animals

Three newborn blind-mole rats captured during several field expeditions (in 2018, 2019 around Carmel mt. area) and three newborn laboratory rats (*Rattus norvegicus*) were used. The animals were anaesthetized with isoflurane overdose and subjected to the primary cells’ isolation protocol described below. Animals experiments were approved by the Institutional Ethics Committee (Reference #316/14, 420/16 and 671/19).

### 4.3. Cell Culture

Primary skin cells were isolated from the animals’ underarm skin region according to previously described method [28]. The primary cells were cultured in DMEM-F12 medium supplemented with 5% fetal bovine serum (FBS) under ambient humidified atmosphere (5% of CO_2_ and 95% of air) at 37 °C On their 2nd passage cells were plated into six-well plates (5 × 10^5^ cells/well) for the targeted metabolomics experiment (for six technical replicates) or into 100 mm culture dishes (1 × 10^6^ cells/dish) for the quantitative PCR assay.

### 4.4. Metabolomic Analysis

The culture medium was removed and replaced for the next 12 h with serum-free (SFM) Dubellco’s Modified Eagle Medium-F12, glucose- and Gln-free (Gibco) supplemented with bovine serum albumin/insulin/transferrin; 2.5 mg/L of ascorbic acid phosphate; 1 mg/L of glutathione; 0.0003 mg/L of ammonium metavanadate; 0.25 nM of manganous chloride; 0.1 mM of acetate; 5 mMg of glucose; 0.6 mM of Gln. 

Two hrs. prior to the beginning of the experiment, freshly-prepared medium with labeled ^13^C_5_ glutamine [Glutamine (U-13C5, 99%)-Cambridge Isotope Laboratories, USA, CLM-1822-H-0.25] (the same composition to SFM a with the non-labelled glutamine replaced by 0.6 mM of ^13^C_5_ glutamine), as well as PBS for control were placed under 1% of O_2_ atmosphere for deoxygenation. The cells were transferred to the hypoxic chamber, the medium removed, and after PBS washing, it was replaced with deoxygenated medium containing ^13^C_5_ glutamine. Similar manipulations were performed for the normoxic conditions in a regular CO_2_ incubator (~20% O_2_). The cells were supplied with labelled ^13^C_5_ glutamine, exposed for 24 hrs. under normoxic (20% of O_2,_ normoxia), and hypoxic (1% of O_2_, hypoxia) in the hypoxic chamber (HypOxystation^®^ H35, HypOxygen, Frederick, MD, USA) conditions. After incubation, culture medium was collected, cells were washed twice with ice-cold PBS and intracellular metabolites were extracted using methanol/acetonitrile/water solution (5:3:2) on the rocker shaker (10 min at 4 °C). The extracts and medium samples were centrifuged (16,000× *g* 10 min, 4 °C), the supernatants (medium samples after 1:50 dilution with mobile phase) were transferred to −80 °C and stored until subjected to LC-MS analysis as described in [94].

### 4.5. qPCR Assay 

RNA was isolated from rat and *Spalax* skin fibroblasts passages 2–3 cultivated under normal or hypoxic conditions (1% oxygen, 24 h) using standard TRI-reagent protocol. RNA concentration and extraction quality were assessed by Epoch microplate spectrophotometer (BioTek, Winooski, VT, USA). For cDNA synthesis iScript cDNA synthesis kit (Bio Rad, Hercules, CA, USA) was used. 1 mg of total RNA was used for 20 µL cDNA. 

Primers for RT qPCR were designed in Primer Express 3.0.1 software (Applied Biosystems, Waltham, MA, USA). Separate pairs of primers were designed for *Spalax* and rat RNA.

RT qPCR reactions were performed by using Fast SYBR Green master mix protocol (Applied Biosystems, USA).1 µL of the cDNA was used for each well (diluted to 5 µL with dH_2_O to ensure accurate pipetting). Serial dilutions of cDNA were used for calibration curve building for the quantification of *Spalax* and rat ALDH4, ALDH18, PYCR and PRODH genes. The reactions were performed in StepOnePlus Real-Time PCR System (Applied Biosystems) and the data was obtained and analyzed by StepOne software v2.3 (Applied Biosystems). The subsequent statistical analysis and graph plotting was made in Microsoft Excel software pack.

### 4.6. Statistical Analysis

Raw data were normalized and *p* values were calculated with GraphPad Prizm 8 software using unpaired Student’s *t*-test (*p* < 0.05 was considered to be statistically significant as specified in the figures legend). 

## 5. Conclusions

The metabolic patterns of most terrestrial mammals, which evolved to function in the regular oxygen atmosphere (~20% O_2_), are characterized by tight and obligatory coupling of Glc and Gln carbon fluxes in the TCA cycle. This system works excellently when all components are available; however, a temporary shortage of oxygen or one of the two nutrients may cause a failure. Oxygen is the keystone of this system; Glc and Gln starvation can be rescued via gluconeogenesis, glycogenosis, or proteolysis, whereas hypoxia can be tolerated only for short periods. The essential challenge for the function of oxidative carbon metabolism under low oxygen conditions is the obligatory coupling of the anaerobic glycolysis and the mitochondrial OXPHOS. Mild and short hypoxic conditions are effectively resolved by HIF family of transcription factors that reprogram metabolism to adapt to oxygen shortage via upregulation of anaerobic glycolysis, decreasing flux of Glc carbons to the TCA cycle and slowdown OXPHOS. Hypoxic HIF-1α-reprogrammed metabolism causes outcomes such as lactic acidosis, specific ROS productions, increased lipid peroxidation, energy shortage, changing redox state, etc., and may lead to apoptotic death of the cell. Nearly 60 years ago, Otto Warburg described the metabolic frame, named Warburg effect [95], that helps several cancer cells to tolerate severe and continuous hypoxia by uncoupling the ATP synthesis from ETC and depending on glycolysis as an energy provider [96]. The ideal metabolic machinery that effectively functions under varying oxygen concentrations must be less dependent on HIF-1α regulations and process cytosolic and mitochondrial carbon fluxes independently or semi-independently. Apparently, *Spalax*’s metabolic adaptation strategy meets these requirements. Gln metabolism in *Spalax* cells mainly focuses on the Glu domain, where the flux of Gln-originated carbon switches between GSH and protein production instead of being processed by TCA cycle reactions. The major rerouting of Gln flux to protein production, putatively collagen, was observed in normoxic and increased in hypoxic *Spalax* cells. The enormous forwarding of Glu to protein production in *Spalax* cell under both normoxia and hypoxia can be considered as storage of reduced Gln carbons and suggests its involvement in bioenergetics and signaling via cycling in Pro shuttle which is HIF-1α-independent. Increased conversion of αKG to 2HG prevents degradation of HIF-1α in hypoxic *Spalax* cells, and thus maintains cytosolic and mitochondrial carbon fluxes uncoupled via inhibition of PDH and suggests specific bioenergetics model that relies on HIF-1α-independent/less dependent coupling to Glc carbons flux. Strong antioxidant defense in *Spalax* cells is attributed to massive use of Glu for GSH production. 

In summary, hypoxic *Spalax* cells employ similar to described under normoxia metabolic frame for distribution of Gln-derived carbons. The upregulation of Gln flux to collagen synthesis and 2HG production are essential metabolic signatures of hypoxic *Spalax* cells that are involved in bioenergetics, antioxidant and epigenetic adaptive strategies to face harsh environmental challenges. 

## Data Availability

Data is contained within the article and supplementary material.

## Supplementary Materials

The following are available online at www.mdpi.com/article/10.3390/metabo11110755/s1, Figure S1: Mass isotoplogues distribution of *Spalax* and rat cells, Figure S2: Selected characteristics of ^13^C_5_ tracing in *Spalax* and rat cells, levels of selected intracellular metabolites, and mRNA expression of selected genes in *Spalax* liver tissues, Figure S3: Selected mitochondrial fluxes of Gln-originated carbons in *Spalax* and rat skin cells, Figure S4: The levels of selected non-labelled metabolites, Figure S5: The levels of mRNA expression of some genes of *proline shuttle* enzymes in *Spalax* and rat liver tissues and skin fibroblasts.
