# Supplementary figures and images for: Glutamine Homeostasis and Its Role in the Adaptive Strategies of the Blind Mole Rat, Spalax
Source: Metabolites. 2021 Oct 31;11(11):755. doi: 10.3390/metabo11110755 (PMC8620300; doi:10.3390/metabo11110755)

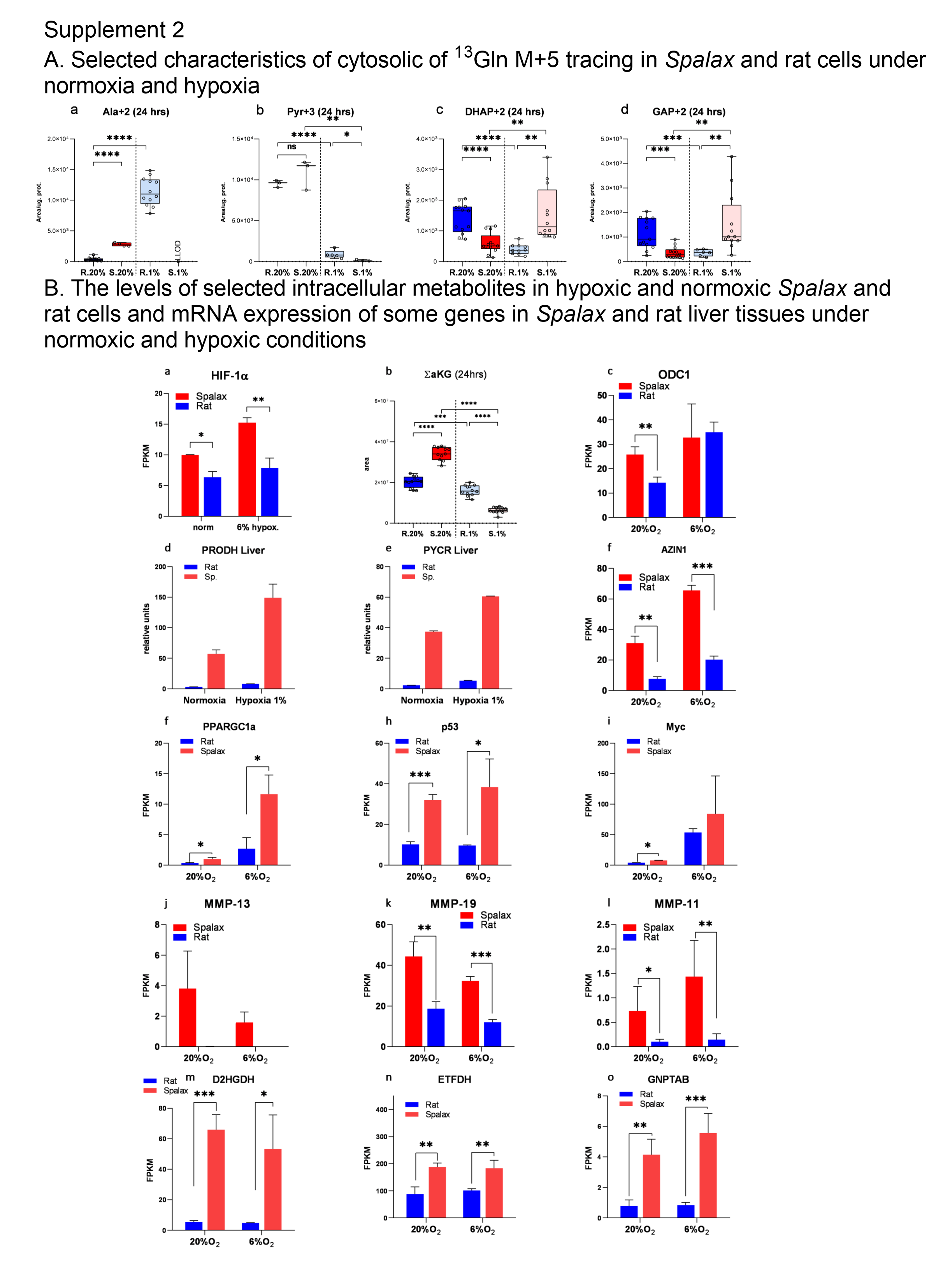

Supplement: Supplementary file 1 [file metabolites-11-00755-s001.zip › S 2.tif]

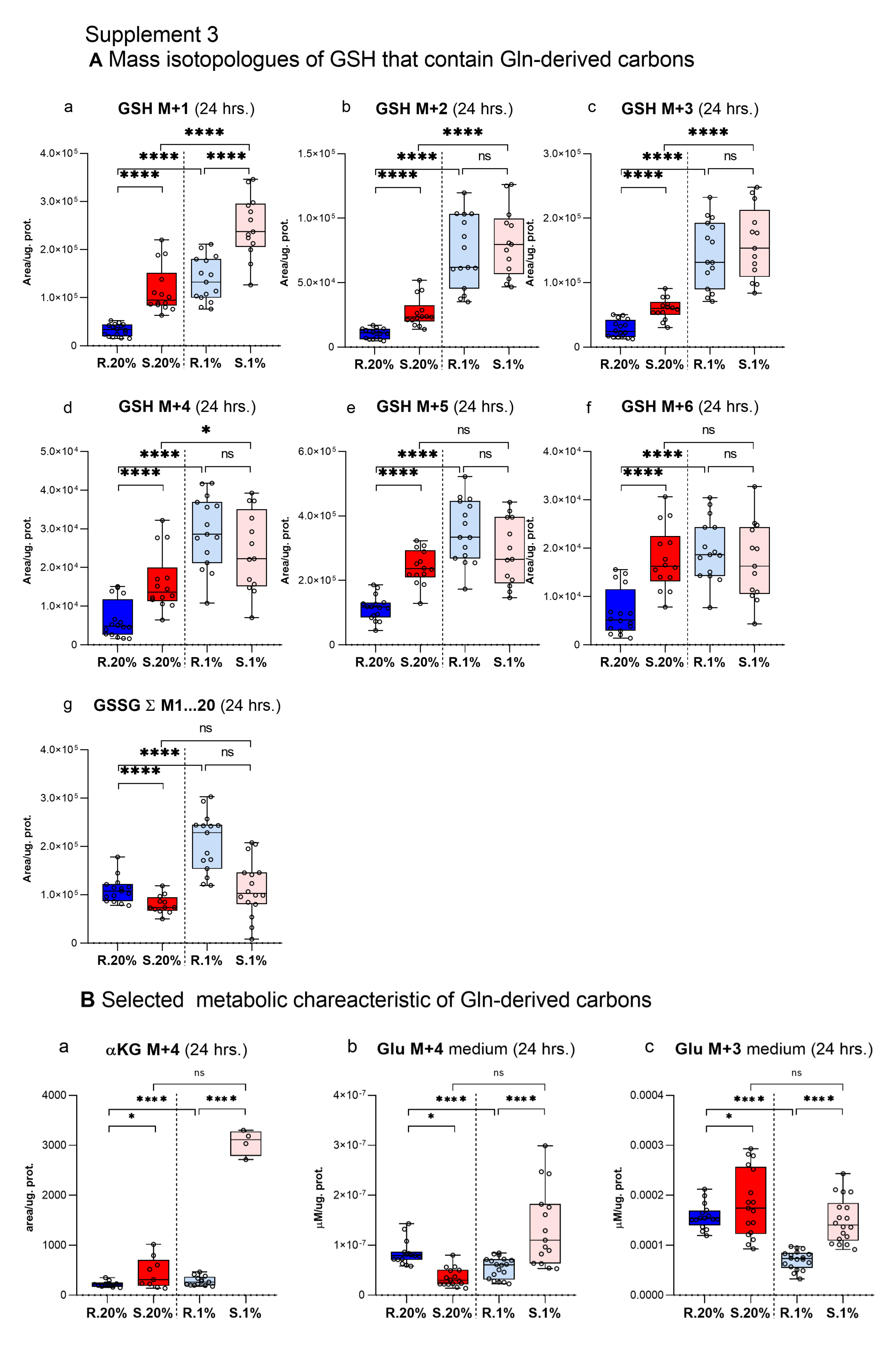

Supplement: Supplementary file 1 [file metabolites-11-00755-s001.zip › S 3.tif]

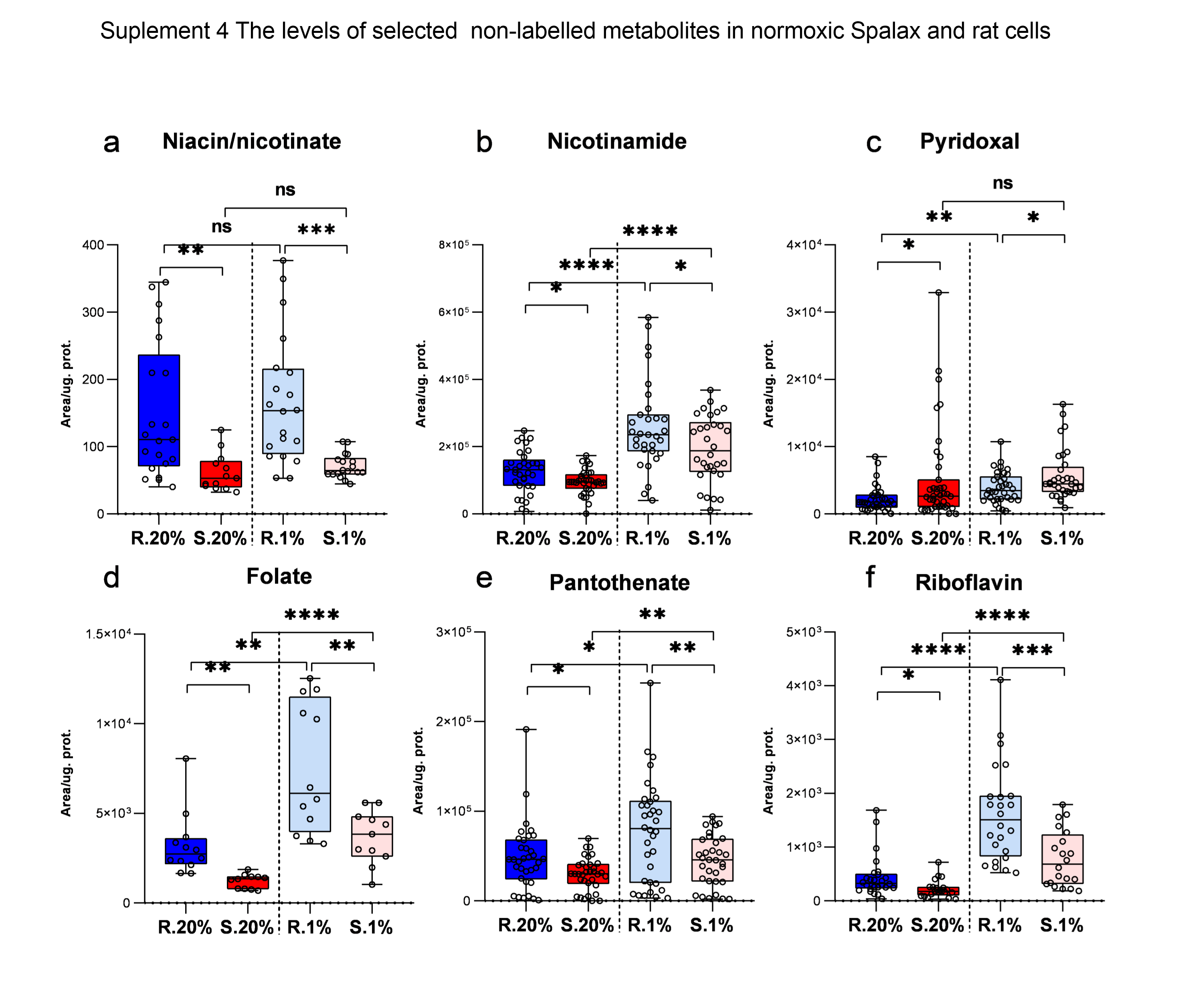

Supplement: Supplementary file 1 [file metabolites-11-00755-s001.zip › S 4.tif]

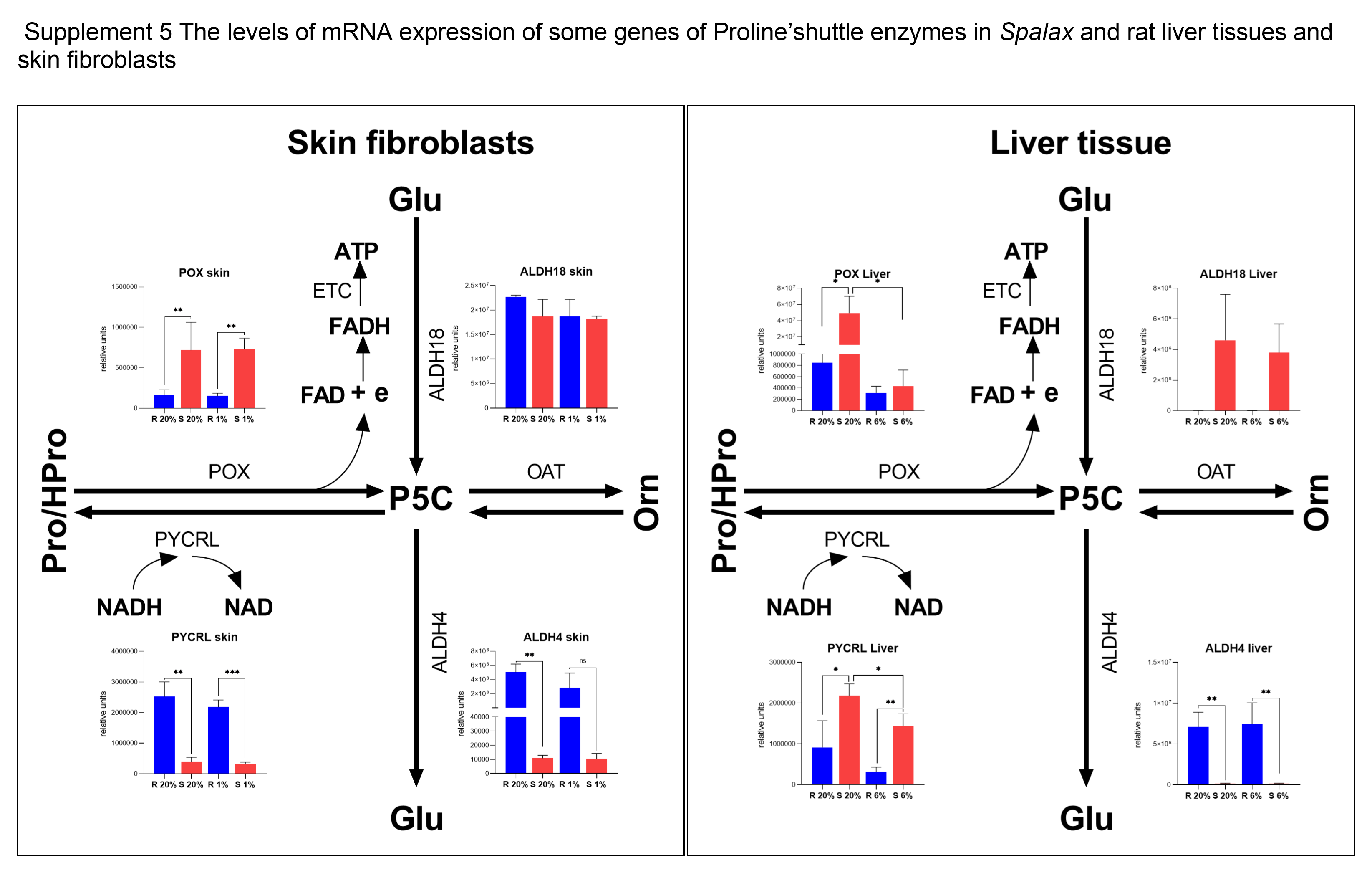

Supplement: Supplementary file 1 [file metabolites-11-00755-s001.zip › S 5.tif]

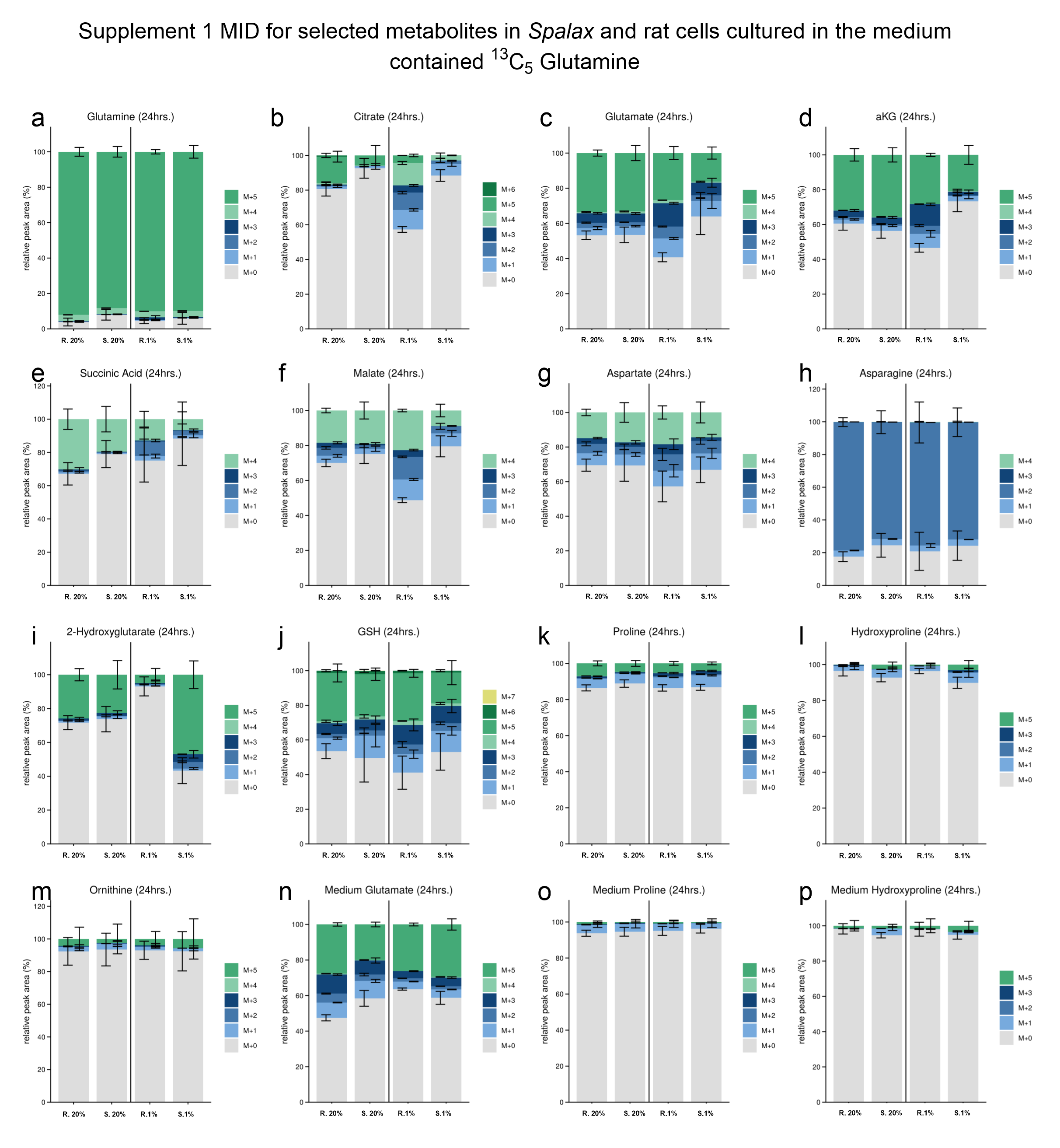

Supplement: Supplementary file 1 [file metabolites-11-00755-s001.zip › S1.tif]
